# Association between Breakfast Frequency and Atherosclerotic Cardiovascular Disease Risk: A Cross-Sectional Study of KNHANES Data, 2014–2016

**DOI:** 10.3390/ijerph16101853

**Published:** 2019-05-25

**Authors:** Hyeon Ji Lee, Jieun Jang, Sang Ah Lee, Dong-Woo Choi, Eun-Cheol Park

**Affiliations:** 1 Department of Public Health, Graduate School, Yonsei University, Seoul 03722, Korea; leehj612@yuhs.ac (H.J.L.); JIEUN99@yuhs.ac (J.J.); CDW6027@yuhs.ac (D.-W.C.); 2 Institute of Health Services Research, Yonsei University, Seoul 03722, Korea; 3 Research and Analysis Team, National Health Insurance Service Ilsan Hospital, Goyang 10444, Korea; IVORY0817@yuhs.ac; 4 Department of Preventive Medicine, Yonsei University College of Medicine, Seoul 03722, Korea

**Keywords:** cardiovascular disease, breakfast, meals, lifestyle, public health

## Abstract

The burden of cardiovascular disease (CVD) is increasing worldwide and one related lifestyle choice is breakfast consumption. This study examined the association between breakfast frequency and the 10-year risk of atherosclerotic CVD (ASCVD). The 10-year risk of ASCVD was defined as the risk of the first event of nonfatal myocardial infarction, coronary heart disease death, and nonfatal or fatal stroke within 10 years. Data from the Korean National Health and Nutrition Examination Survey, 2014–2016 were analyzed, and 7212 participants aged 40–79 years with no history of CVD were included. ASCVD risk was calculated according to the pooled cohort ASCVD equation, and participants with a score >7.5% were considered at high risk of ASCVD. The association between breakfast frequency and high ASCVD risk was confirmed by logistic regression analysis. Participants who never ate breakfast were more likely to be in the high-risk group compared to participants who ate breakfast >5 times per week (OR (adjusted odds ratio) = 1.46; 95% CI (confidence interval) = 1.12–1.89), and the risk was especially higher in female participants and those with a family history of CVD. Our study confirms that breakfast consumption even once per week may prevent CVD.

## 1. Introduction

Cardiovascular disease (CVD), including heart attack and stroke, has a high mortality rate worldwide [1]. In South Korea, an estimated 53,597 deaths occurred from CVD in 2017, making it the second most common cause of mortality, accounting for 19% of all deaths in the country [2]. Additionally, in 2016, the medical cost of CVD was reported to be about 8 trillion Korean won (approximately 7 billion US dollars) [3]. Obesity, hypertension, diabetes, and dyslipidemia are the main causes of CVD [4] and the increasing prevalence of these diseases in adults in modern society raises valid concerns regarding a drastic increase in the incidence of CVD in the future. However, many of the diseases that lead to CVD are lifestyle-related and can be prevented. 

Accordingly, various methods for identifying groups at high risk of CVD have been proposed, with the aim of implementing lifestyle interventions and to reduce the number of CVD-related deaths [5]. In 2013, the American College of Cardiology/American Heart Association (ACC/AHA) Guidelines for Cardiovascular Risk Assessment proposed the concept of the “pooled cohort atherosclerotic cardiovascular disease (ASCVD) equation,” which partly addresses the limitations of previous methods, and assesses a 10-year ASCVD risk [6,7].

The lifestyle choices associated with CVD are smoking, drinking, reduced exercise, sleeping patterns, and eating patterns or habits [8]. Among these factors, modern society is becoming more aware of eating habits, and as a result, many people are conscientious about the nutritional content and quality of their meals [9,10]. However, the importance of timing and frequency of meals are often overlooked. Breakfast is associated with more health effects than lunch or dinner [10]. Breakfast consumption enables diverse nutrient intake and addresses energy imbalance, is helpful for weight loss and weight management, and is associated with reduced risk of developing various diseases, such as CVD, diabetes, and dyslipidemia [10]. In addition, eating breakfast is closely linked to a healthier lifestyle [11]. Therefore, eating breakfast is an important health promotion determinant. The National Health Promotion Strategy (Health Plan 2020) in Korea includes the goal of reducing the rate of breakfast skipping [12] because although it is important for health, many people do not regularly eat breakfast.

According to the 4th National Health Promotion Strategies (Health Plan 2020) [12], a survey of people aged 1 year and over, the breakfast skipping rate in Korea in 2013 was 22.5%, and the target rate for 2020 is 18.3%. However, according to the Korea National Health and Nutrition Examination Survey (KNHANES) for adults aged 19 years and over, the breakfast skipping rate has been gradually increasing since 2013 (25.2% in 2013, 25.6% in 2014, 28.0% in 2015, and 29.6% in 2016) [13]. Such a trend can also be observed in other countries [14,15,16].

Many studies, both national and international, have examined the relationship between breakfast skipping and health status. Several studies have shown that breakfast skipping has an impact on depression [17], obesity, diabetes, hypertension, metabolic disease [18,19], and CVD [20,21,22,23]. A cohort study in the United States showed that the risk of coronary heart disease increases by an average of 27% in men who skip breakfast [20], and a Japanese cohort study showed that men who skipped breakfast had a significantly increased risk of mortality from both circulatory disease and all causes [21]. Two studies evaluated the effects of breakfast frequency on CVD risk and categorized breakfast frequency into 3 groups (0–2, 3–5, or 6–7 times per week) [22] and 4 groups (0–2, 3–4, 5–6, or 7 times per week) [23]. Together, these studies indicated that eating breakfast less than three times (0–2 times) per week was associated with a high risk of CVD. Therefore, our study aimed to expand these findings by investigating the difference between groups of individuals that always skip breakfast and groups that eat breakfast at least once per week.

Ultimately, the purpose of this study was to examine the association between the frequency of breakfast skipping per week and the 10-year risk of ASCVD. Additionally, we determined the risk of CVD in people who eat breakfast once a week and those do not eat breakfast at all. Therefore, the groups of individuals from the KNHANES study were subdivided into those who ate breakfast 0, 1–2, 3–4, and 5–7 times per week.

## 2. Materials and Methods

### 2.1. Data Collection and Participants

This cross-sectional study used data from the 2014–2016 KNHANES, which included a total of 23,080 respondents. We excluded individuals with a diagnosis of CVD (*n* = 809; including stroke, myocardial infarction, and angina), individuals aged <40 years or >79 years (*n* = 12,810) because the ASCVD risk equation is specific for the age range 40–79 years, individuals with unknown breakfast frequency (*n* = 1059), and individuals who had not provided information regarding age, sex, socioeconomic status (SES), health-related factors, and survey year (*n* = 1197). Finally, a total of 7,205 individuals were included in this study. The KNHANES data are openly published; thus, ethical approval was not required for this study. Additionally, information of respondents was fully anonymized and unidentified before analysis. Therefore, this study did not require informed consent from the respondents.

### 2.2. Dependent Variable

The main outcome for this study was the assessment of ASCVD risk. The 10-year risk of ASCVD was defined as the risk of the first event of nonfatal myocardial infarction, coronary heart disease death, and nonfatal or fatal stroke within 10 years [6]. The risk of CVD was assessed by applying the pooled cohort risk assessment equation from the 2013 ACC/AHA guidelines [6]. The 10-year ASCVD risk score considers factors of age; sex; ethnicity; high-density lipoprotein cholesterol (HDL) and total cholesterol (TC) levels; systolic blood pressure (SBP; whether hypertension is treated or not); the prevalence of diabetes; and smoking status. Since it is appropriate for Koreans to be considered as Caucasians, regression coefficients for the white race were applied to the equation in this study. Participants with a risk score of >7.5% were regarded as at high risk because this score is used to indicate whether a prescription of statins should be considered [6]. In order to identify absolute risk, the dependent variable was designed in the form of a binary division with a cut-off of 7.5% (i.e., high-risk group: ≥7.5%, normal group: <7.5%).

### 2.3. Independent Variable

The independent variable in this study was the frequency at which breakfast was eaten per week. The questionnaire about the frequency of meals per day in the KNHANES is divided into breakfast, lunch, and dinner, which could be defined according to when one eats. Breakfast can be defined as a meal taken in the morning [24]. The following KNHANES questionnaire item was used to determine the independent variable, “How many times per week did you have breakfast in the last year?” There are four possible responses to the question: 5–7 times per week, 3–4 times per week, 1–2 times per week, almost none (0 times per week). Participants were categorized according to their responses to this questionnaire item.

### 2.4. Control Variables

Covariates were age, sex, socioeconomic status (SES), health-related factors, and survey year. SES included marital status, education level, household income level, occupation (white collar, pink collar, blue collar, and others), and rural/urban region. Health-related factors were alcohol status, perceived stress level, perceived health status, physical activity, body mass index (BMI), nutrition status, total energy, fat and carbohydrate intake per day, and family history of CVD. Physical activity was categorized into 3 groups, considering the amount and intensity of physical activity according to the metabolic equivalent task (MET). MET was calculated by considering strength, time, and the number of days of physical activity (MET range <600, low; 600–2999, moderate; ≥3000, high) [25]. Family history was categorized as ‘Yes’ if the respondent indicated they had a family history of CVD (including stroke, myocardial infarction, or angina) and ‘No’ if they did not. Nutrition status was categorized by mean nutrient adequacy ratio [MAR(9)]. MAR(9) calculates the nutrient adequacy ratio (NAR) of nine nutrients (protein, calcium, phosphorus, iron, vitamin A, vitamin B1, vitamin B2, niacin, and vitamin C), adds them all together, and divides by nine [26]. A MAR(9) score of >0.75 was categorized as good and a score <0.75 was categorized as poor [27].

### 2.5. Statistical Analysis

The chi-square test, *t*-test, and logistic regression were used for the analysis of the data. The chi-square test was used to examine the significance of differences in the ASCVD risk by the frequency of breakfast after adjusting for covariates. *p*-values <0.05 were considered significant. The association and significance of differences between ASCVD risk (dependent variable) and the number of breakfasts per week (independent variable) were analyzed with logistic regression analysis for adjusted odds ratios (ORs) and 95% confidence intervals (CIs). Subgroup analysis was performed by sex, MET, and family history. In addition, we tested for multicollinearity in this statistical model. The variance inflation factors (VIFs) were all less than 10, indicating no excessive correlation between independent variables. All statistical analyses were performed using SAS software, version 9.4 (SAS Institute Inc., Cary, NC, USA).

## 3. Results

### 3.1. Study Population

The general characteristics of the study population are provided in Table 1. The total number of participants was 7205, of which 2786 were considered ASCVD high-risk, and 4419 were considered ASCVD normal. In both the high-risk and normal groups, the frequency of breakfast consumption was 5–7 per week for most participants. With the exception of survey year (*p* = 0.7889), the differences in all other variables between ASCVD risk groups were statistically significant. In the ASCVD high-risk group, participants were most likely to eat breakfast 5–7 times per week (43.2%; *n* = 2414), followed by 0 times per week (28.5%; *n* = 169).

### 3.2. Association between Breakfast Frequency and ASCVD Risk

Table 2 shows the association between variables and ASCVD high-risk. Logistic regression analyses adjusted for covariates revealed that breakfast frequency was related to ASCVD risk. Individuals who did not eat breakfast (0 times per week) were significantly more likely to belong to the high-risk group than those who ate breakfast 5–7 times per week (reference group) (OR = 1.46; 95% CI = 1.12–1.89). The risk for women was lower than that for men (OR = 0.06; 95% CI = 0.05–0.07). A family history of CVD had no statistical impact on ASCVD risk (OR = 0.88; 95% CI = 0.75–1.03).

### 3.3. Association between ASCVD High-Risk and Breakfast Frequency Stratified by Sex and Family History

**Figure 1:**
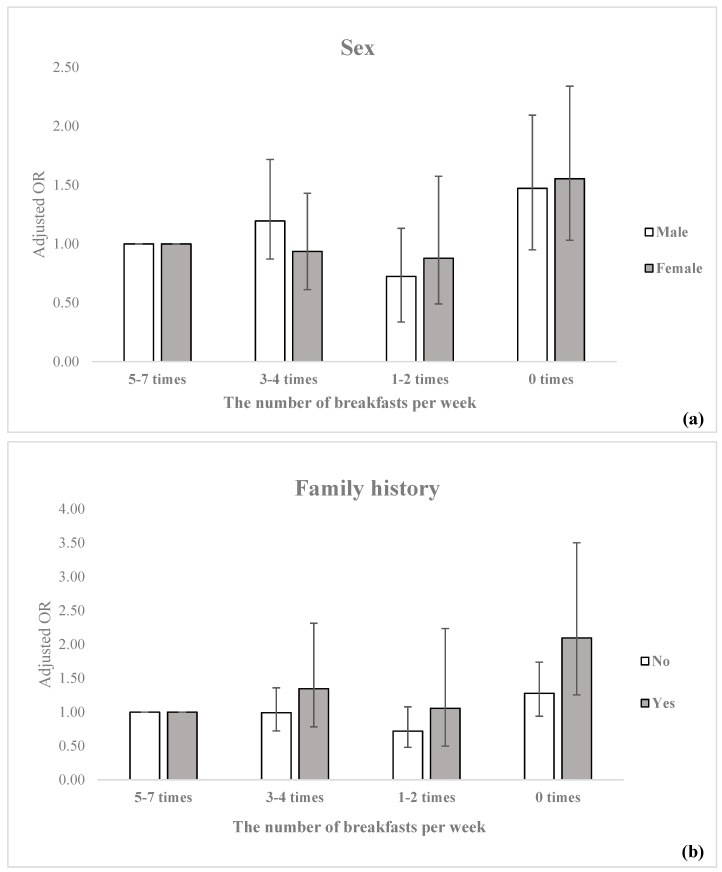
Subgroup analysis of the association between ASCVD high-risk and breakfast frequency stratified by (**a**) sex and (b) family history. Adjusted OR, all covariates were adjusted. ASCVD risk: 10-year ASCVD risk was measured using a pooled cohort equation. 10-year risk <7.5%: normal group, ≥7.5%: high-risk group. (**b**) Family history: related to CVD such as stroke, myocardial infarction, angina. ASCVD: atherosclerotic cardiovascular disease, adjusted OR: adjusted odds ratio.

Figure 1 shows the results of subgroup analyses regarding the impact of sex and family history on ASCVD high-risk according to the number of breakfasts consumed per week. Both men and women who did not eat breakfast were more likely to be ASCVD high-risk compared to the 5–7 times reference group (men: OR = 1.47, 95% CI = 1.04–2.09; women: OR = 1.55, 95% CI = 1.03–2.34), with women having a higher likelihood of being in the high-risk group compared to men. Compared to individuals without a family history of CVD, those who reported a family history of CVD tended to have higher ORs for high ASCVD risk when the frequency of breakfast consumption was ≤4 times per week; in particular, individuals who did not eat breakfast at all were significantly more likely to be at high risk (OR = 2.10; 95% CI = 1.26–3.50).

## 4. Discussion

This study examined the association between the number of times breakfast was eaten per week and the risk of ASCVD incidence in 10 years determined through the pooled cohort ASCVD equation, using nationally representative KNHANES data. Our results indicate that individuals who regularly fail to eat breakfast are statistically significantly more likely to belong to the high-risk group for ASCVD than those who eat breakfast more than 5 times per week. This result was similar for both sexes. Additionally, women who involve in intense physical activity or those with a family history of CVD were more likely to be at high-risk if they consistently skip breakfast.

A previous cross-sectional study on the relationship between the breakfast frequency and CVD incidence for middle-aged Korean men divided breakfast frequency into 3 categories: hardly ever (0–2 times per week), sometimes (3–5 times per week), and always (6–7 times per week). The study found that the “hardly ever” group had significantly higher triglyceride levels [22]. Another similar study using Japanese cohort data divided breakfast frequency into 4 categories (0–2, 3–4, 5–6, and 7 times per week) and showed that the incidence of CVD was significantly higher in groups that ate breakfast 0–2 times per week compared to groups that ate breakfast every day [23]. Together, these studies indicate that people who rarely eat breakfast (mainly less than 3 times per week) have a higher risk of CVD than those who regularly eat breakfast.

Our study found that women had a higher CVD risk than men when the frequency of breakfast consumption was ≤2 times per week. Many previous studies have reported that women who do not eat breakfast have health problems linked to CVD-related factors such as high TC or low-density lipoprotein-cholesterol, elevated blood pressure, low cortisol, and obesity [28,29,30,31,32,33]. In addition, some studies have reported more relevance to obesity when skipping breakfast in women than men [28,29,30]. A previous study has underscored the importance of preventing and managing CVD in women for the following reasons: lower attention to CVD due to past perceptions that CVD is a disease predominantly prevalent among males, and higher obesity rates in women than in men [34]. Our study supports the importance of CVD management in women.

Our study results support and extend findings from previous studies by indicating that eating breakfast once or twice per week can help prevent CVD compared to not eating breakfast at all. Breakfast is eaten at the beginning of the day and a healthy day that begins with breakfast may improve an individuals’ overall healthy lifestyle. A previous study suggested that skipping breakfast can have an impact on other meals during the day [35] and can reflect an individual’s general healthy behavior [10,11]. The present study is meaningful in terms of public health because it considers the preventative aspects of CVD, which has a high disease burden in modern societies. Moreover, it clarifies the association between CVD and breakfast frequency, a lifestyle habit that can be modified for disease prevention. The strengths of the study included the fact that it used a large, national data set compiled by national institutions, increasing the reliability of the original data. In addition, the study excluded individuals previously diagnosed with cardiovascular-related diseases to minimize the weaknesses of a cross-sectional study design.

However, several limitations should be taken into account when interpreting the results of this study. First, because this was a cross-sectional study, the results cannot identify a definite causal relationship; because breakfast is not an intervention, the relatively low risk of CVD in individuals who eat breakfast more often may be related to their generally healthier lifestyles. Second, despite the efforts of the surveying agency to reduce bias, the original data we analyzed may have been affected by response bias. In addition, the KNHANES data regarding the frequency of meals were obtained based on the timing of the meal (i.e., breakfast, lunch, and dinner) so the frequency of meals could be checked according to the meal constitution. However, it was not possible to determine the items consumed for breakfast, since nutritional data were only given for total daily intake and the corresponding nutritional content. Therefore, we considered the total intake of nutritional factors per day as control variables. Future studies need to identify the exact amount of calories and nutrients intakes for breakfast. Third, a 10-yr ASCVD risk assessment model (pooled cohort ASCVD equation) from the ACC/AHA guideline was used to estimate the risk of ASCVD in the study sample. The guideline is suitable for non-Hispanic Caucasians or African-Americans, the risk may be over-estimated for Asians. However, since this is a problem that is based on patients diagnosed with ASCVD, its use can also be tolerated in Asians from a preventive perspective [36]. In addition, an appropriate model for Asians has not been developed. Thus in many Asian countries, including South Korea, a 10-yr ASCVD risk assessment model has been used [37,38,39,40,41,42,43]. Finally, because the pooling cohort ASCVD equation is only applicable to individuals aged 40–79 years, this study could not assess the CVD risk for other age groups.

## 5. Conclusions

The results of this study suggest that daily breakfast skipping is associated with an increased risk of CVD, and indicates that eating breakfast even once per week may prevent CVD. In particular, the results highlight the role of a family history of CVD in CVD risk in women. Furthermore, the results emphasize the importance of eating breakfast and suggests a simple lifestyle change of eating breakfast at least once per weak that can reduce the incidence of CVD and prevent ASCVD.

## Figures and Tables

**Table 1:** General characteristics of the study population.

Variables	Total		ASCVD Risk				*p*-Value
			High-Risk		Normal		
	*n*	%	*n*	%	*n*	%	
**Breakfasts per week**							<0.0001
5–7 times	5589	77.6	2,414	43.2	3175	56.8	
3–4 times	597	8.3	137	22.9	460	77.1	
1–2 times	425	5.9	66	15.5	359	84.5	
0 times	594	8.2	169	28.5	425	71.5	
**Sex**							<0.0001
Male	2916	40.5	1726	59.2	1190	40.8	
Female	4289	59.5	1060	24.7	3229	75.3	
**Age**							<0.0001
40-49	2158	30.0	154	7.1	2004	92.9	
50-59	2189	30.4	524	23.9	1665	76.1	
>60	2858	39.7	2108	73.8	750	26.2	
**Marital status**							<0.0001
Married	5930	82.3	2164	36.5	3766	63.5	
Single, separated, or divorced	1275	17.7	622	48.8	653	51.2	
**Educational level**							<0.0001
Middle school or less	2757	38.3	1545	56.0	1212	44.0	
High school	2385	33.1	699	29.3	1686	70.7	
College or over	2063	28.6	542	26.3	1521	73.7	
**Household income level**							<0.0001
Low	1298	18.0	823	63.4	475	36.6	
Lower middle	1810	25.1	796	44.0	1014	56.0	
Upper middle	1909	26.5	577	30.2	1332	69.8	
High	2188	30.4	590	27.0	1598	73.0	
**Occupation**							<0.0001
White collar	1415	19.6	316	22.3	1099	77.7	
Pink collar	1008	14.0	203	20.1	805	79.9	
Blue collar	2033	28.2	957	47.1	1076	52.9	
Unemployed or other	2749	38.2	1310	47.7	1439	52.3	
**Region**							<0.0001
Urban area	4472	62.1	1647	36.8	2825	63.2	
Rural area	2733	37.9	1139	41.7	1594	58.3	
**Alcohol status**							<0.0001
Non-drinker	1003	13.9	480	47.9	523	52.1	
Other	6202	86.1	2306	37.2	3896	62.8	
**Perceived stress level**							<0.0001
Low	5608	77.8	2284	40.7	3324	59.3	
High	1597	22.2	502	31.4	1095	68.6	
**Perceived health status**							0.0016
Good	2116	29.4	821	38.8	1295	61.2	
Normal	3752	52.1	1394	37.2	2358	62.8	
Bad	1337	18.6	571	42.7	766	57.3	
**Physical activity**							0.0001
High	1173	16.3	393	33.5	780	66.5	
Moderate	2990	41.5	1158	38.7	1832	61.3	
Low	3042	42.2	1235	40.6	1807	59.4	
**BMI (kg/m^2^)**							<0.0001
Underweight or Normal (<22.9)	2810	39.0	953	33.9	1857	66.1	
Overweight (23.0–24.9)	1861	25.8	768	41.3	1093	58.7	
Obesity (>25.0)	2534	35.2	1065	42.0	1469	58.0	
**Family history**							0.0010
No	5539	76.9	2199	39.7	3340	60.3	
Yes	1666	23.1	587	35.2	1079	64.8	
**Nutritional status**							<0.0001
Good	5284	73.3	1924	36.4	3360	63.6	
Poor	1921	26.7	862	44.9	1059	55.1	
Calorie intake (kcal/day) *	1973.6	861.5	1995.1	853.5	1960.0	866.3	<0.0001
Fat intake (g/day) *	39.5	31.6	35.4	29.2	42.1	32.8	<0.0001
Carbohydrate intake (g/day) *	313.7	127.7	325.7	133.1	306.0	123.6	<0.0001
**Year**							0.7889
2014	2203	30.6	850	38.6	1353	61.4	
2015	2481	34.4	972	39.2	1509	60.8	
2016	2521	35.0	964	38.2	1557	61.8	
Total	7205	100.0	2786	38.7	4419	61.3	

* Values are presented as mean ± standard deviation. *p*-value is the result of the chi-square test and *t*-test; ASCVD risk: 10-year ASCVD risk was measured using the pooled cohort equation; 10-year risk <7.5%: normal group, ≥7.5%: high-risk group; ASCVD: atherosclerotic cardiovascular disease; BMI: body mass index.

**Table 2:** Association between the number of breakfasts per week and high risk of ASCVD.

Variables	Total	
	Adjusted OR *	95% CI
**Breakfasts per week**		
5–7 times	1.00	-
3–4 times	1.06	(0.81–1.39)
1–2 times	0.78	(0.55–1.11)
0 times	1.46	(1.12–1.89)
**Sex**		
Male	1.00	-
Female	0.06	(0.05–0.07)
**Age**		
40–49	0.02	(0.01–0.02)
50–59	0.09	(0.08–0.11)
>60	1.00	-
**Marital status**		
Married	1.00	-
Single, separated, or divorced	1.50	(1.26–1.78)
**Educational level**		
Middle school or less	1.49	(1.19–1.88)
High school	1.21	(0.99–1.49)
College or over	1.00	-
**Household income level**		
Low	1.61	(1.29–2.03)
Lower middle	1.15	(0.94–1.40)
Upper middle	0.98	(0.81–1.19)
High	1.00	-
**Occupation**		
White collar	1.00	-
Pink collar	0.83	(0.63–1.11)
Blue collar	0.98	(0.77–1.25)
Unemployed or other	1.56	(1.22–1.99)
**Region**		
Urban area	1.00	-
Rural area	1.11	(0.96–1.28)
**Alcohol status**		
Non–drinker	1.00	-
Other	0.76	(0.63–0.92)
**Perceived stress level**		
Low	1.00	-
High	0.97	(0.83–1.15)
**Perceived health status**		
Good	1.00	-
Normal	1.01	(0.86–1.19)
Bad	0.80	(0.65–0.99)
**Physical activity**		
High	1.00	-
Moderate	1.22	(0.99–1.50)
Low	1.40	(1.14–1.72)
**BMI (kg/m^2^)**		
Underweight or Normal (<22.9)	1.00	-
Overweight (23.0–24.9)	1.11	(0.93–1.32)
Obesity (>25.0)	1.19	(1.02–1.40)
**Family history**		
No	1.00	-
Yes	0.88	(0.75–1.03)
**Nutritional status**		
Good	1.00	-
Poor	1.18	(0.99–1.41)
**Calorie intake (kcal/day)**	1.00	(1.00–1.00)
**Fat intake (g/day)**	1.00	(1.00–1.00)
**Carbohydrate intake (g/day)**	1.00	(0.99–1.00)
**Year**		
2014	1.04	(0.88–1.23)
2015	0.98	(0.83–1.16)
2016	1.00	-

* OR: adjusted odds ratio. All covariates were adjusted; ASCVD risk: 10-year ASCVD risk was measured using the pooled cohort equation; 10-year risk <7.5%: normal group, ≥7.5%: high-risk group; ASCVD: atherosclerotic cardiovascular disease, adjusted OR: odds ratio, 95% CI: confidence interval; BMI: body mass index.

## References

[1] World Health Organization World Heart Day 2017. http://www.who.int/cardiovascular_diseases/world-heart-day-2017/en/.

[2] Korean Statistical Information Service Cause of death statistics. http://kosis.kr/statHtml/statHtml.do?orgId=101&tblId=DT_1B34E01&vw_cd=MT_ZTITLE&list_id=D11&seqNo=&lang_mode=ko&language=kor&obj_var_id=&itm_id=&conn_path=MT_ZTITLE#.

[3] Son M., Seong S. (2016). National Health Insurance Statistical Yearbook.

[4] Mokdad A.H., Ford E.S., Bowman B.A., Dietz W.H., Vinicor F., Bales V.S., Marks J.S. (2003). Prevalence of obesity, diabetes, and obesity-related health risk factors, 2001. JAMA.

[5] Damen J.A., Hooft L., Schuit E., Debray T.P., Collins G.S., Tzoulaki I., Lassale C.M., Siontis G.C., Chiocchia V., Roberts C. (2016). Prediction models for cardiovascular disease risk in the general population: Systematic review. BMJ.

[6] Goff D.C., Lloyd-Jones D.M., Bennett G., Coady S., D’Agostino R.B., Gibbons R., Greenland P., Lackland D.T., Levy D., O’Donnell C.J. (2014). 2013 ACC/AHA guideline on the assessment of cardiovascular risk: A report of the American College of Cardiology/American Heart Association Task Force on Practice Guidelines. J. Am. Coll. Cardiol..

[7] Muntner P., Colantonio L.D., Cushman M., Goff D.C., Howard G., Howard V.J., Kissela B., Levitan E.B., Lloyd-Jones D.M., Safford M.M. (2014). Validation of the Atherosclerotic Cardiovascular Disease Pooled Cohort Risk EquationsCardiovascular Disease Risk EquationsCardiovascular Disease Risk Equations. JAMA.

[8] World Health Organization (2007). Prevention of Cardiovascular Disease.

[9] Kant A.K., Schatzkin A., Graubard B.I., Schairer C. (2000). A prospective study of diet quality and mortality in women. JAMA.

[10] O’Neil C.E., Byrd-Bredbenner C., Hayes D., Jana L., Klinger S.E., Stephenson-Martin S. (2014). The role of breakfast in health: Definition and criteria for a quality breakfast. J. Acad. Nutr. Diet..

[11] Keski-Rahkonen A., Kaprio J., Rissanen A., Virkkunen M., Rose R.J. (2003). Breakfast skipping and health-compromising behaviors in adolescents and adults. Eur. J. Clin. Nutr..

[12] Ministry of Health and Welfare (2015). Korea Health Promotion Foundation. Health Plan 2020 (2016–2020).

[13] Korea Centers for Disease Control and Prevention (2017). Korea Health Statistics 2016: Korea National Health and Nutrition Examination Survey (KNHANES Ⅶ-1).

[14] O’Neil C.E., Nicklas T.A., Fulgoni V.L. (2014). Nutrient intake, diet quality, and weight/adiposity parameters in breakfast patterns compared with no breakfast in adults: National Health and Nutrition Examination Survey 2001–2008. J. Acad. Nutr. Diet..

[15] Drewnowski A., Rehm C., Vieux F. (2018). Breakfast in the United States: Food and Nutrient Intakes in Relation to Diet Quality in National Health and Examination Survey 2011–2014. A Study from the International Breakfast Research Initiative. Nutrients.

[16] Haines P.S., Guilkey D.K., POPKIN B. (1996). Trends in breakfast consumption if US adults between 1965 and 1991. J. Am. Diet. Assoc..

[17] Lee S.A., Park E.-C., Ju Y.J., Lee T.H., Han E., Kim T.H. (2017). Breakfast consumption and depressive mood: A focus on socioeconomic status. Appetite.

[18] Odegaard A.O., Jacobs D.R., Steffen L.M., Van Horn L., Ludwig D.S., Pereira M.A. (2013). Breakfast frequency and development of metabolic risk. Diabetes Care.

[19] Yoo K.-B., Suh H.-J., Lee M.-J., Kim J.-H., Kwon J.A., Park E.-C. (2014). Breakfast eating patterns and the metabolic syndrome: The Korea National Health and Nutrition Examination Survey (KNHANES) 2007–2009. Asia Pac J Clin Nutr..

[20] Cahill L.E., Chiuve S.E., Mekary R.A., Jensen M.K., Flint A.J., Hu F.B., Rimm E.B. (2013). Prospective study of breakfast eating and incident coronary heart disease in a cohort of male US health professionals. Circulation.

[21] Yokoyama Y., Onishi K., Hosoda T., Amano H., Otani S., Kurozawa Y., Tamakoshi A. (2016). Skipping breakfast and risk of mortality from cancer, circulatory diseases and all causes: Findings from the Japan Collaborative Cohort Study. Yonago Acta Med..

[22] Yim K.S. (2000). Effects of Lifestyle and Dietary Behavior on Cardiovascular Risks in Middle-aged Korean Men. J. Community Nutr..

[23] Kubota Y., Iso H., Sawada N., Tsugane S., Group J.S. (2016). Association of breakfast intake with incident stroke and coronary heart disease: The Japan Public Health Center-Based Study. Stroke.

[24] Jung C.-H., Lee J.S., Ahn H.J., Choi J.-S., Noh M.Y., Lee J.J., Lee E.Y., Lim J.H., Lee Y.R., Yoon S.Y. (2017). Association of meal frequency with metabolic syndrome in Korean adults: From the Korea National Health and Nutrition Examination Survey (KNHANES). Diabetol. Metab. Syndr..

[25] Ashok P., Kharche J.S., Raju R., Godbole G. (2017). Metabolic equivalent task assessment for physical activity in medical students. Natl. J. Physiol. Pharm. Pharmacol..

[26] Kim H.R. (2016). Quality of Diet and Nutritional Intake and Mortality Risk among South Korean Adults Based on 12-year Follow-up Data. Korean J. Community Nutr..

[27] Lee J.S., Kim H.Y., Hwang J.Y., Kwon S., Chung H.R., Kwak T.-K., Kang M.H., Choi Y.S. (2018). Development of Nutrition Quotient for Korean adults: Item selection and validation of factor structure. J. Nutr. Health.

[28] Barr S.I., DiFrancesco L., Fulgoni V.L. (2015). Association of breakfast consumption with body mass index and prevalence of overweight/obesity in a nationally-representative survey of Canadian adults. Nutri. J..

[29] Kant A.K., Andon M.B., Angelopoulos T.J., Rippe J.M. (2008). Association of breakfast energy density with diet quality and body mass index in American adults: National Health and Nutrition Examination Surveys, 1999–2004. Am. J. Clin. Nutr..

[30] Song W.O., Chun O.K., Obayashi S., Cho S., Chung C.E. (2005). Is consumption of breakfast associated with body mass index in US adults?. J. Am. Diet. Assoc..

[31] Mohiuddin A. (2019). Skipping Breakfast Everyday Keeps Well-Being Away. J. Dairy Sci. Technol..

[32] Witbracht M., Keim N.L., Forester S., Widaman A., Laugero K. (2015). Female breakfast skippers display a disrupted cortisol rhythm and elevated blood pressure. Physiol. Behav..

[33] Deshmukh-Taskar P., Nicklas T.A., Radcliffe J.D., O’Neil C.E., Liu Y. (2013). The relationship of breakfast skipping and type of breakfast consumed with overweight/obesity, abdominal obesity, other cardiometabolic risk factors and the metabolic syndrome in young adults. The National Health and Nutrition Examination Survey (NHANES): 1999–2006. Public Health Nutr..

[34] Mosca L., Barrett-Connor E., Kass Wenger N. (2011). Sex/gender differences in cardiovascular disease prevention: What a difference a decade makes. Circulation.

[35] Levitsky D.A., Pacanowski C.R. (2013). Effect of skipping breakfast on subsequent energy intake. Physiol. Behav..

[36] Chia Y.C., Lim H.M., Ching S.M. (2014). Validation of the pooled cohort risk score in an Asian population—A retrospective cohort study. BMC Cardiovasc. Disord..

[37] Park S.Y., Chin S.O., Rhee S.Y., Oh S., Woo J.-T., Kim S.W., Chon S. (2018). Cardio-ankle vascular index as a surrogate marker of early atherosclerotic cardiovascular disease in Koreans with type 2 diabetes mellitus. Diabetes Metab. J..

[38] Cho Y.G. (2018). Cardiovascular Risk Prediction in Korean Adults. Korean J. Fam. Med..

[39] Wu X., Du R., Hu C., Cheng D., Ma L., Li M., Xu Y., Xu M., Chen Y., Li D. (2019). Resting heart rate is associated with metabolic syndrome and predicted 10-Year risk of cardiovascular disease: A cross-sectional study. J. Diabetes.

[40] Lee K. (2017). Muscle Mass and Body Fat in Relation to Cardiovascular Risk Estimation and Lipid-Lowering Eligibility. J. Clin. Densitom..

[41] Kim M.H., Kim Y.S., Oh H.J., Kwon Y.R., Kim H.W. (2018). The association between 10-year atherosclerotic cardiovascular diseases risk score calculated using 2013 American College of Cardiology/American Heart Association guidelines and serum 25-hydroxyvitamin D level among aged 40–79 years in Korea: The sixth Korea National Health and Nutrition Examination Surveys. Korean J. Fam. Med..

[42] Lin L., Long W., LIU S.S., ZHAO Z.Y., Mian L., WANG T.G., Min X., LU J.L., CHEN Y.H., WANG S.Y. (2019). Association between Coronary Atherosclerotic Plaque Composition and Cardiovascular Disease Risk. Biomed. Environ. Sci..

[43] Satoh-Asahara N., Kotani K., Yamakage H., Yamada T., Araki R., Okajima T., Adachi M., Oishi M., Shimatsu A., Group M.S.S.J. (2015). Cardio-ankle vascular index predicts for the incidence of cardiovascular events in obese patients: A multicenter prospective cohort study (Japan Obesity and Metabolic Syndrome Study: JOMS). Atherosclerosis.

